# Identification of circRNA expression signatures correlated with disease severity in pediatric systemic lupus erythematosus

**DOI:** 10.3389/fimmu.2025.1608509

**Published:** 2025-07-31

**Authors:** Feng Li, Huishan Chen, Ying Tang, Suyun Cheng, Ping Zeng, Yiqian Wang

**Affiliations:** ^1^ Department of Allergy, Immunology and Rheumatology, Guangzhou Women and Children’s Medical Center, Guangzhou Medical University, Guangzhou, China; ^2^ GMU-GIBH Joint School of Life Sciences, The Guangdong-Hong Kong-Macao Joint Laboratory for Cell Fate Regulation and Diseases, Guangzhou Medical University, Guangzhou, China

**Keywords:** circRNA, RNA sequencing, pediatric systemic lupus erythematosus, biomarker, disease activity

## Abstract

**Background:**

Systemic lupus erythematosus (SLE) is a complex systemic autoimmune disease with no current cure. Developing diagnostic biomarkers is crucial for improving patient outcomes. Circular RNAs (circRNAs) are a class of noncoding RNAs that are more stable, abundant, and structurally distinct compared to linear RNAs. While circRNAs have shown promise as biomarkers in various diseases, their potential in pediatric SLE remains unclear.

**Methods:**

We performed RNA sequencing on peripheral blood mononuclear cells (PBMCs) from pediatric SLE patients categorized into mild, moderate, and severe groups. CircRNA expression profiles were analyzed for differential expression. The potential of circRNAs as biomarkers for SLE severity was evaluated through receiver operating characteristic (ROC) analysis. Additionally, Spearman correlation analysis was used to assess the relationship between circRNA expression levels and the SLE Disease Activity Index (SLEDAI) score.

**Results:**

Our analysis revealed significant differential expression of circRNAs across different SLE severity groups. The circRNA expression patterns were closely associated with various biological processes, including signaling pathways, metabolism, and transcriptional regulation. Furthermore, ROC analysis demonstrated the potential of circRNAs to predict SLE severity. Spearman correlation analysis showed a significant correlation between dysregulated circRNA expression and SLEDAI scores.

**Conclusion:**

Our findings strongly suggest that circRNAs could play a pivotal role in predicting pediatric SLE severity, offering a promising avenue for early diagnosis and personalized treatment strategies. This research lays the groundwork for future studies exploring circRNAs in pediatric SLE pathogenesis and prognosis, with the potential to significantly improve patient outcomes and therapeutic interventions.

## 1 Introduction

Systemic Lupus Erythematosus (SLE) is a chronic autoimmune disease that can affect multiple organ systems, including the skin, joints, kidneys, heart and nervous system (1, 2). SLE is characterized by the production of autoantibodies that target various cellular components, leading to inflammation and tissue damage (1, 3). Notably, children represent approximately 15% to 20% of all SLE patients and typically experience more severe disease at onset compared to adults (4). Furthermore, SLE is more common in females, particularly those of reproductive age, and is often associated with genetic, environmental, and hormonal factors (5). Treatment for SLE primarily involves non-specific anti-inflammatory and immunosuppressive medications, including antimalarial drugs, glucocorticoids (GCs), non-corticosteroid immunosuppressants, and targeted therapies (6). Although the prognosis and survival rates for SLE patients have significantly improved over the past decade, the disease remains characterized by an unpredictable and fluctuating course, with periods of relapse and remission spanning many years (7). Therefore, identifying more effective biomarkers for prognosis and potential therapeutic targets in SLE is crucial for overcoming chemotherapy resistance and improve clinical outcomes.

Circular RNAs (circRNAs) are a recently identified class of non-coding RNA (ncRNAs), which form a special covalent closed-loop structure, lacking both a 5’ to 3’ direction and a polyadenylated tail (8). Numerous studies have demonstrated that circRNAs are multifunctional molecules involved in regulating gene expression at both transcriptional and post-transcriptional levels, influencing normal physiological processes as well as disease conditions (9). In recent years, circRNAs have gained attention as valuable biomarkers for clinical diagnosis and prognosis due to their high stability, abundance, and distinct expression patterns (10). Dysregulation of circRNAs has been reported in various types of cancer, including breast cancer, lung cancer, and colorectal cancer (11). For example, increased expression of upregulated circRNAs is associated with poor prognosis in non-small cell lung cancer (NSCLC) (12). Additionally, the circRNA circRPPH1 has been shown to promote breast cancer progression via the circRPPH1-miR-512-5p-STAT1 axis (13). Additionally, circRNA has also been reported as potential biomarkers for the diagnosis and severity of autoimmune diseases, such as rheumatoid arthritis (RA), multiple sclerosis (MS) and primary biliary cholangitis (PBC) (14). A previous study demonstrated that CircRNA_09505 exacerbates joint damage in collagen-induced arthritis mice by regulating the miR-6089/AKT1/NF-κB signaling pathway (15). The dysregulation of hsa_circ_402458 plays a role in the pathogenesis of primary biliary cholangitis (PBC), highlighting its potential as a biomarker for the disease (16). However, the role of circRNAs in SLE is not yet fully understood and warrants further exploration.

In this study, we explored the circRNA expression profile in SLE patients across varying severity groups to investigate its potential as a prognostic indicator for the disease. KEGG and GO analyses further highlighted potential pathways regulated by key circRNAs across different severity groups. Thus, our findings provide insights into the molecular mechanisms of circRNAs and highlight their potential role in predicting the prognosis of SLE.

## 2 Materials and methods

### 2.1 Patients

Peripheral blood mononuclear cells (PBMCs) were obtained from a total of 18 pediatric patients diagnosed with SLE at Guangzhou Women and Children’s Medical Center, Guangzhou Medical University (Guangzhou, China). Based on the SLE disease activity index (SLEDAI), patients were categorized into the following groups: mild (1–10), moderate (11–15), and severe (16–20). The research was approved by the Ethics Committee of Guangzhou Women and Children’s Medical Center and conducted in accordance with the principles of the Helsinki Declaration (ethics number: 2023-330B00). Consent to participate was obtained from all respondents, and the consent form was signed by their adult guardians. Unfortunately, detailed information on treatment regimens and disease duration was not consistently available and thus not included.

### 2.2 Sample collection and total RNA extraction

Blood samples were collected after an overnight fast. A total of 2 mL of blood was drawn from the median cubital vein of each subject and stored in EDTA anticoagulant vacutainers. Total RNA was extracted within 4 hours using TRIzol^®^ reagent (Invitrogen, Inc.; Thermo Fisher Scientific, Inc.) following the manufacturer’s protocol. RNA concentration and quality were evaluated by measuring absorbance ratios of A260/A280 and A260/A230 using a NanoDrop ND-1000 spectrophotometer (Agilent Technologies, Inc.).

### 2.3 CircRNA sequencing analysis

#### 2.3.1 Quality control

Raw Data are processed using Perl scripts to ensure the quality of data for subsequent analysis. The following filter criteria have been applied:

1. Adaptor-polluted reads are filtered out, defined as reads containing more than 5 adapter-polluted bases. For paired-end sequencing data, both reads of a pair are excluded if either of the paired-end reads is contaminated with adapters.
2. Filter out low-quality reads, defined as those where more than 15% of bases have a Phred quality score of 19 or lower.
3. Filter out reads in which the proportion of N bases exceeds 5%.

For paired-end sequencing data, both reads from a pair are filtered out if either read fails to meet the criteria outlined above. The clean data is generated after filtering, and statistical analyses are conducted to evaluate its quantity and quality, including Q30 statistics, data volume metrics, base composition, and other pertinent parameters.

#### 2.3.2 Mapping the sequencing reads to reference genomes

The reference genomes and annotation files were downloaded from the ENSEMBL database (http://www.ensembl.org/index.html). Reads were aligned to the reference genome using the BWA-MEM method, a fast and efficient approach that facilitates the mapping of fragmented reads to the genome.

#### 2.3.3 Quantitation of gene expression

The expression level of circRNA is measured by junction reads, represented as SRPBM (Spliced Reads per Billion Mapped).


$$SRPBM=SRx10^{9}/N$$

Where SR represents the number of spliced reads, and N denotes the total number of mapped reads in a given sample.

#### 2.3.4 Differential expression analysis

Differential expression analysis of two samples, with or without replicates, was performed using DEGseq (http://www.bioconductor.org/packages/release/bioc/html/DEGseq.html) or DESeq (http://www.bioconductor.org/packages/release/bioc/html/DESeq.html). Assuming that the read counts for a gene (or transcript isoform) follow a binomial distribution, DEGseq, based on the MA-plot, is commonly employed for such analyses. A *P*-value is calculated for each gene, which is then adjusted for multiple comparisons using the Benjamini-Hochberg (BH) method. Genes with a q-value < 0.05 and |log2 FC| ≥ 1 are considered differentially expressed.

#### 2.3.5 GO and KEGG pathway enrichment analysis

To assess whether genes are associated with a specific Gene Ontology (GO) term (http://geneontology.org/), a hypergeometric *P*-value is calculated and adjusted to a q-value, with the background set to all genes in the genome. GO terms with a q-value < 0.05 are considered significantly enriched, indicating the biological functions performed by differentially expressed genes (DEGs). Additionally, KEGG (Kyoto Encyclopedia of Genes and Genomes, http://www.kegg.jp/) provides a comprehensive database of manually curated pathway maps that represent molecular interaction and reaction networks. Significantly enriched KEGG pathways are identified using the same method applied in the GO enrichment analysis.

### 2.4 Statistical analysis

Data are presented as the mean ± standard deviation (SD) and were analyzed using GraphPad Prism 10.0. The number of biological replicates (n) is indicated in the figure legends. Statistical comparisons were made using the Student’s *t*-test, with *P* < 0.05 considered statistically significant. For circRNA-seq data, false discovery rates (FDR) were calculated to adjust *P*-values for multiple testing, ensuring robust results.

## 3 Results

### 3.1 Clinical data of subjects

In this study, pediatric SLE patients were categorized into mild, moderate, and severe groups according to their SLEDAI scores at admission. The values for C3 levels, C4 levels, anti-nuclear antibodies, double-strand (ds) DNA, white blood cell (WBC) count, hemoglobin (Heme), platelet (PLT) count, CD19, erythrocyte sedimentation rate (ESR), 24-hour proteinuria, and urine red blood cell (RBC) count are also shown. The clinical and laboratory characteristics of the patients involved in this study are summarized in **Table 1**. Subsequent analyses focused on comparisons between mild and moderate groups as well as between moderate and severe groups to identify circRNA biomarkers related to disease severity.

**Table 1:** Clinical and laboratory characteristics of systemic lupus erythematosus (SLE) patients involved in this study.

Patient ID	Sex	C3 (g/L)	C4 (g/L)	ANA (IU/L)	dsDNA(IU/L)	WBC (x 10^9^)	Hb (g/L)	PLT (x 10^9^)	CD19 (%)	ESR (mm/h)	24-h proteinuria(g)	Urine RBCs (per μL)	SLEDAI
1	M	0.75	0.15	45	1.25	5.9	131	287	11.43	5	0.11	42	6
2	F	1.14	0.16	0.25	0.27	3.2	119	231	9.64	3	0.52	3.25	5
3	M	0.98	0.24	1.05	0.36	13.6	122	274	8.13	10	0.78	32.56	8
4	F	1.12	0.46	360.55	0.23	7.5	145	221	16.79	33	0.51	25.62	8
5	F	0.69	0.21	2.34	35.23	3.8	93	346	17.44	32	0.06	0	5
6	F	0.68	0.1	2.29	0.78	6.5	136	345	16.57	10	0.98	2.68	6
7	M	0.61	0.12	224.98	66.57	7.9	130	231	22.39	26	1.28	56.2	12
8	F	0.22	0.03	256.23	122.84	3.1	97	213	29.7	9	0.98	78.3	13
9	F	0.5	0.07	189.6	78.69	8.9	100	348	23.63	15	1.02	2.3	10
10	F	0.18	0.06	247.23	105.23	5.2	118	158	27.6	48	0.12	1.2	10
11	F	0.39	0.04	389.63	187.63	3.5	122	253	22.01	25	0.25	11.2	12
12	M	0.55	0.11	22.29	15.21	3.4	126	78	29.36	37	1.56	45.3	11
13	F	0.24	0.04	329.62	268.36	1.2	108	36	34.55	44	2.36	24.3	16
14	F	0.21	0.02	314.57	108.28	1.8	61	125	43.21	89	0.26	11	18
15	M	0.2	0.04	135.36	44.61	2.9	109	337	39.85	47	1.96	69.3	16
16	F	0.14	0.03	289.63	127.7	3.9	102	136	33.05	67	2.55	56.6	16
17	F	0.15	0.12	369.56	154.2	8	57	54	27.53	67	4.8	11.2	20
18	M	0.12	0.03	456.36	259.36	2.6	89	44	39.63	85	0.21	0	16

ANA, anti-nuclear antibodies; WBC, white blood cells; Hb, hemoglobin; C3, complement 3; C4, complement 4; ESR, erythrocyte sedimentation rate.

### 3.2 Differential expression of circRNA in patients with mild and moderate SLE

**Figure 1:**
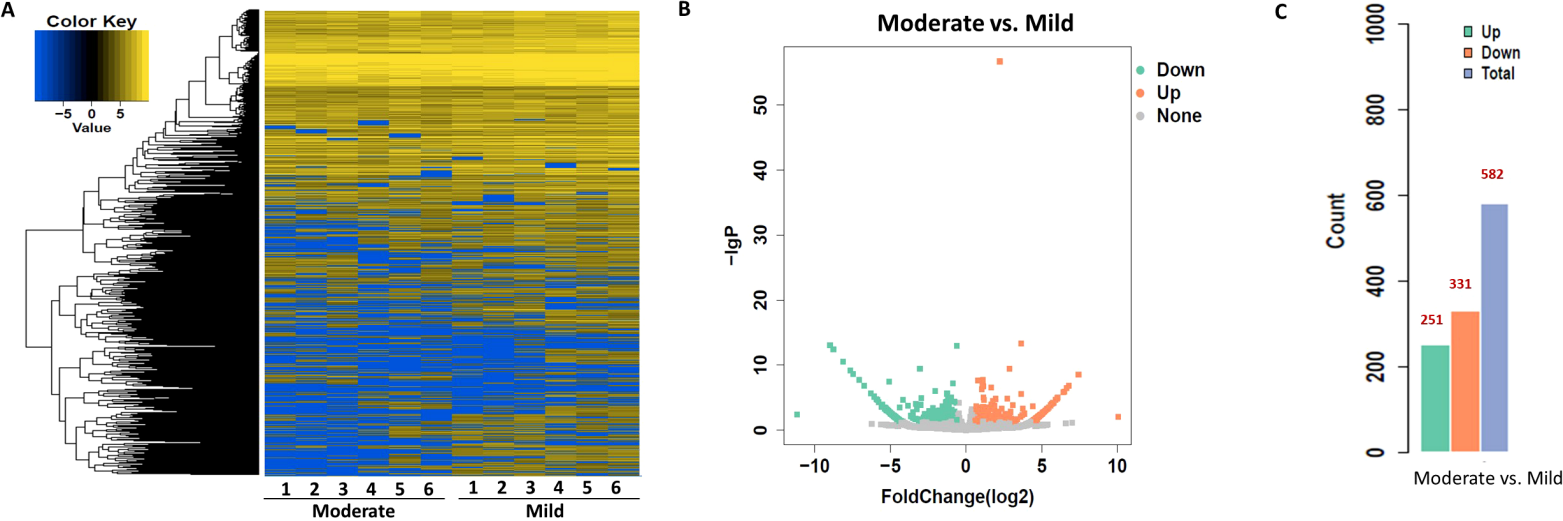
Heatmap and Volcano Plot of differentially expressed circRNAs between mild and moderate SLE groups. **(A)** The heatmap analysis revealed distinct circRNA expression profiles between the two groups. **(B)** The volcano plot shows differentially expressed circRNAs based on RNA-seq analysis. **(C)** The bar graph displays 331 downregulated circRNAs and 251 upregulated circRNAs between the two groups. N=6 each group.

To identify circRNAs associated with progression from mild to moderate SLE, differential expression and bioinformatic analyses were performed comparing six mild and moderate patients. To clarify which circRNAs may serve as biomarkers for the severity of SLE, differential expression and bioinformatic analysis of circRNAs were carried out in six patients with mild and moderate SLE. The fold change (FC) of circRNA expression in both groups was measured to identify differentially expressed circRNAs, as shown in the heatmap (**Figure 1**). A volcano plot identified a total of 582 differentially expressed circRNAs, including 251 upregulated and 331 downregulated circRNAs (**Figures 1B**).

**Figure 2:**
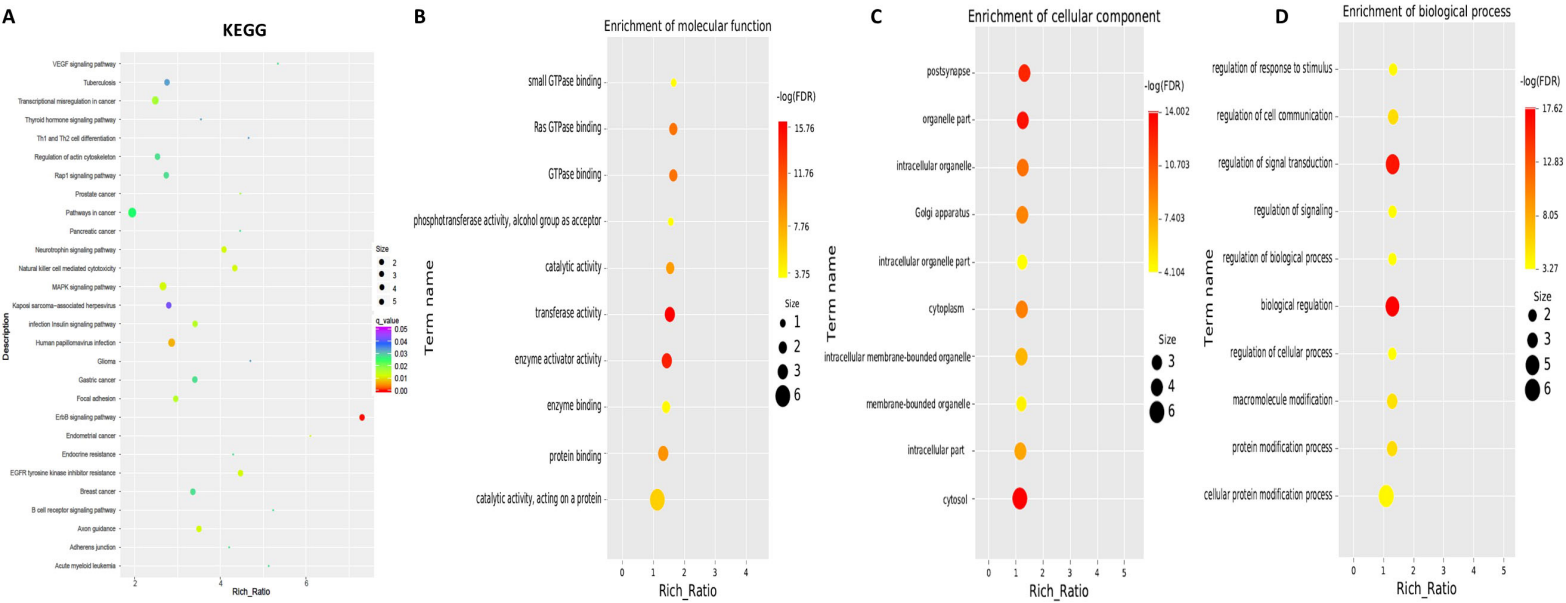
KEGG and GO Pathway enrichment analysis of differentially expressed circRNAs between mild and moderate pediatric SLE patients. **(A)** KEGG pathway enrichment analysis showing the top pathways significantly associated with differentially expressed circRNAs. **(B)** Molecular Function Gene Ontology (GO) terms enriched among the differentially expressed circRNAs. **(C)** Cellular Component GO terms enriched among the differentially expressed circRNAs.**(D)** Biological Process GO terms enriched among the differentially expressed circRNAs.N=6 each group.

To explore the functions associated with dysregulated circRNAs, we performed GO term and KEGG pathway enrichment analyses of circRNA-associated genes between mild and moderate patient groups. KEGG pathway enrichment analysis of circRNA-hosting genes revealed significant differences, identifying 28 pathways that were enriched among the differentially expressed circRNAs. Notably, the ErbB signaling pathway, human papillomavirus infection, and axon guidance were among the pathways significantly enriched in these circRNAs (**Figure 2**, **Supplementary Table S1**). Additionally, Gene Ontology (GO) term enrichment analysis identified 107 terms related to Molecular Function (MF), 47 terms related to Cellular Component (CC), and 332 terms related to Biological Process (BP) in mild and moderate SLE patients. Key enriched terms included catalytic activity, protein binding, enzyme binding and cellular protein modification process (**Figure 2B**, **Supplementary Tables S2**-**S4**). These results suggest that circRNAs may regulate key signaling pathways, enzymatic activities, and protein modification processes involved in the pathogenesis of pediatric SLE. The differential expression patterns of circRNAs are visualized in **Figure 1**, while **Figure 2** focuses on the functional enrichment of these dysregulated circRNAs.

**Figure 3:**
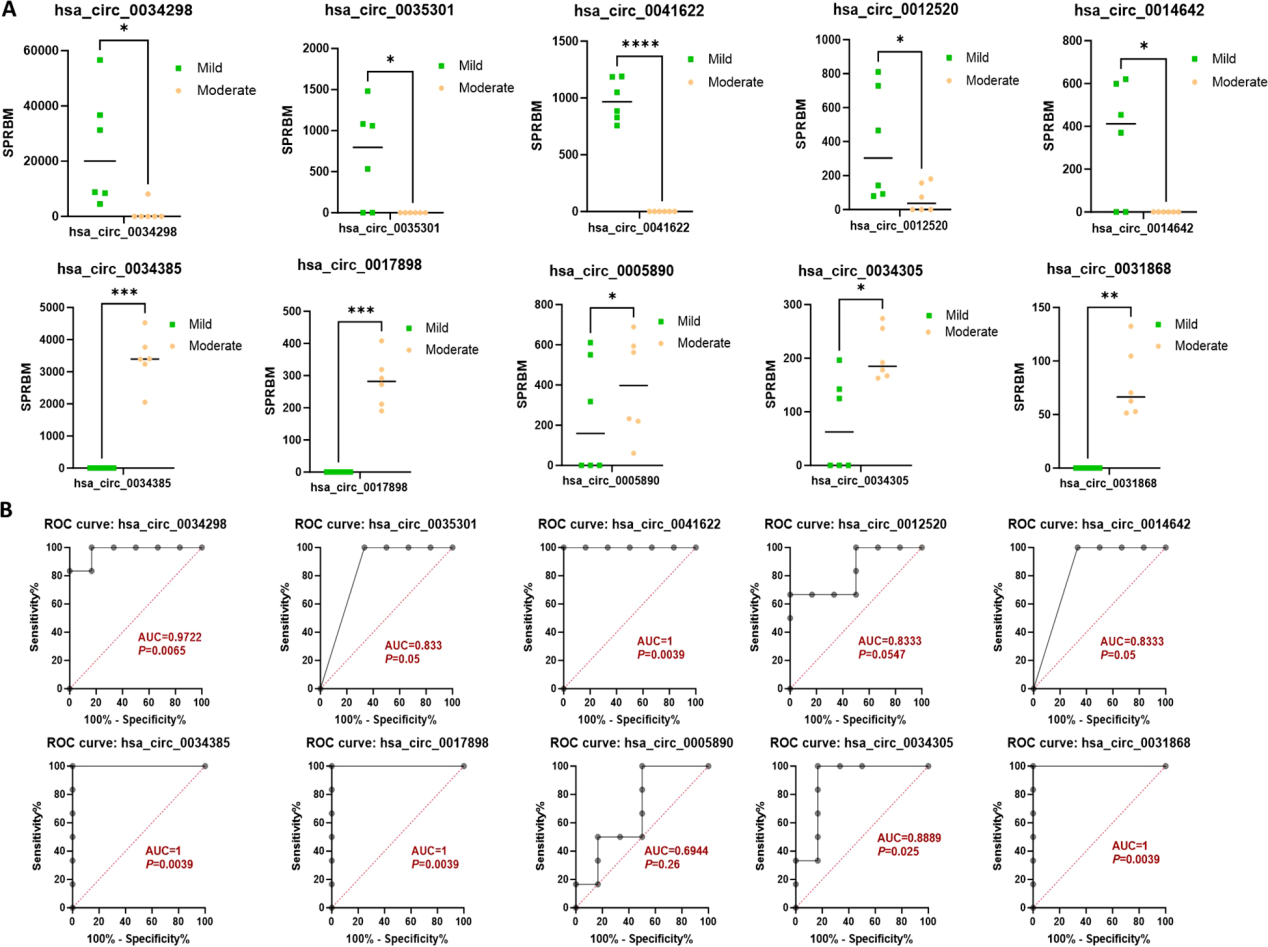
Characterization of differential expressed circRNAs in PBMCs from SLE mild and moderate patients. **(A)** Comparison of expression levels of differentially expressed circRNAs between the two groups. **(B)** ROC curve analysis of differentially expressed circRNAs in the mild and moderate groups, with AUC values displayed. N=6 each group. *P < 0.05; **P < 0.01; ***P < 0.001; ****P < 0.0001.

**Figure 4:**
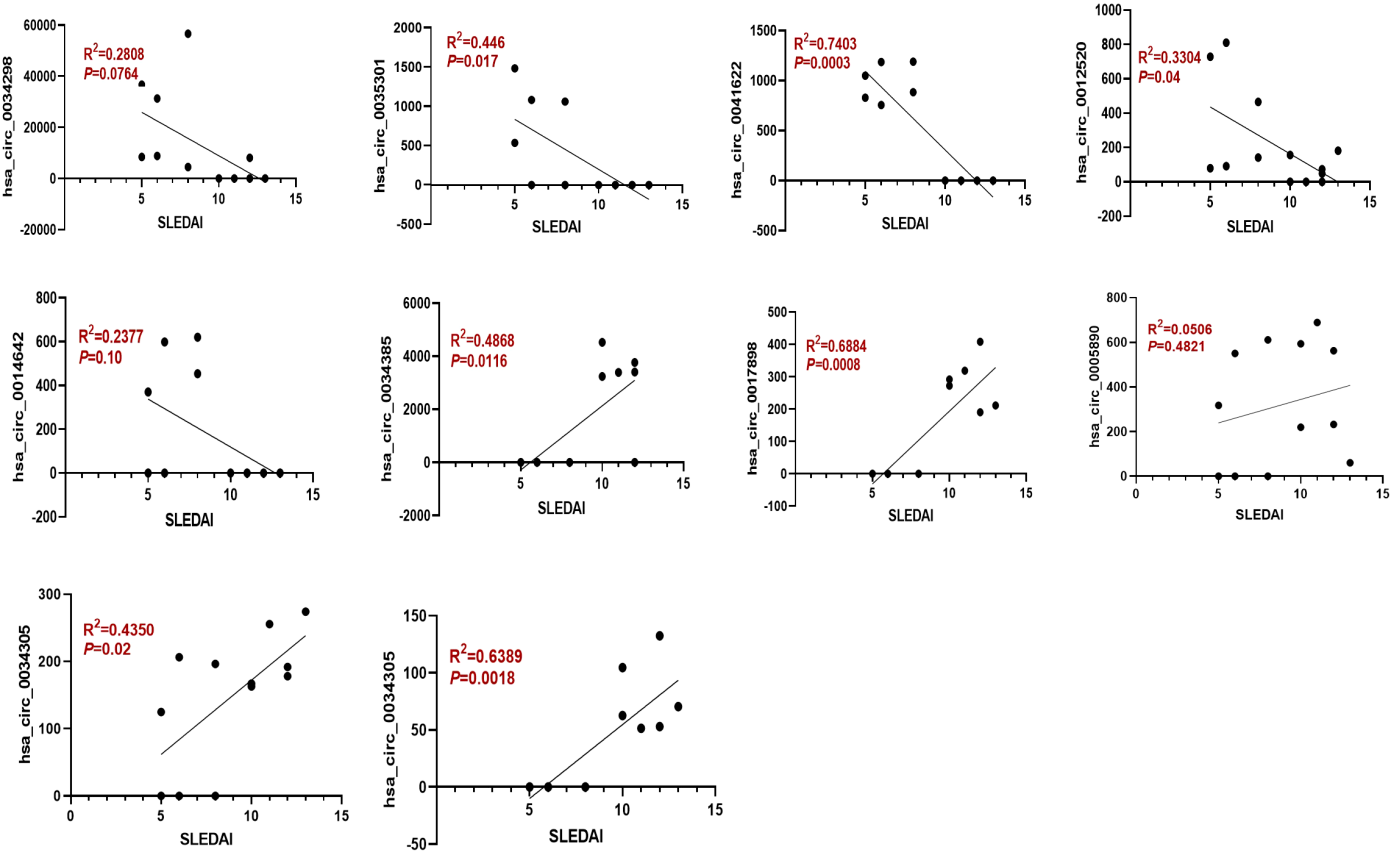
The expression of dysregulated circRNAs is closely related to the SLEDAI Score. Spearman correlation analysis was performed to show the association between differentially expressed circRNAs and SLEDAI scores in mild and moderate SLE patients. N=6 each group.

Moreover, the ten circRNAs with the largest differences (5 up-regulated and 5 down-regulated) based on SPRBM are shown in **Figure 3** and summarized in **Table 2**. To further evaluate the predictive value of differentially expressed circRNAs we identified, receiver operating characteristic (ROC) curves were constructed. The results indicated that most of the identified circRNAs had significant diagnostic value for SLE severity, except for hsa_circ_0035301, hsa_circ_0012520, and hsa_circ_0005890 (**Figure 3**). To further validate the prognostic value of the identified circRNAs, we examined the correlations between circRNA expression and the SLEDAI score. The results showed that the expression levels of most circRNAs were significantly correlated with the SLEDAI score (*P*<0.05). However, the expression of hsa_circ_0034298 and hsa_circ_0014642 did not show any correlation with the SLEDAI score (**Figure 4**). These findings support the hypothesis that circRNAs could serve as useful prognostic markers for distinguishing mild SLE from moderate SLE.

**Table 2:** The top ten circular RNAs (circRNAs) with the most significant expression differences (both upregulation and downregulation) between moderate and mild systemic lupus erythematosus (SLE) patients.

Circ_name	Up/Down	Location	Gene_name	Log2FoldChange	*P* value	*P* adjusted
hsa_circ_0034298	down	6:31271073.31355592:-	Null	-11.17033388	0.004249	0.179032
hsa_circ_0035301	down	7:107121952.107153251:+	PRKAR2B	-8.97648356	9.35E-14	2.25E-10
hsa_circ_0041622	down	Y:19587210.19587507:+	TXLNGY	-8.75845137	3.77E-13	5.94E-10
hsa_circ_0012520	down	17:36182524.36195912:-	Null	-8.111574421	3.14E-11	4.13E-08
hsa_circ_0014642	down	18:62072673.62102902:-	PIGN	-7.630611313	6.89E-10	6.03E-07
hsa_circ_0034385	up	6:35468947.35470335:+	RPL10A	10.06628118	0.009986	0.307746
hsa_circ_0017898	up	1:205616478.205627874:-	ELK4	7.4346598	3.05E-09	2.18E-06
hsa_circ_0005890	up	12:89459638.89472275:-	POC1B	6.802419727	1.62E-07	7.12E-05
hsa_circ_0034305	up	6:31354106.31355590:+	Null	6.64449698	3.26E-07	0.000113
hsa_circ_0031868	up	5:176975396.176982623:-	UIMC1	6.632870154	3.31E-07	0.000113

### 3.3 Differential expression of circRNA in patients with moderate and severe SLE

**Figure 5:**
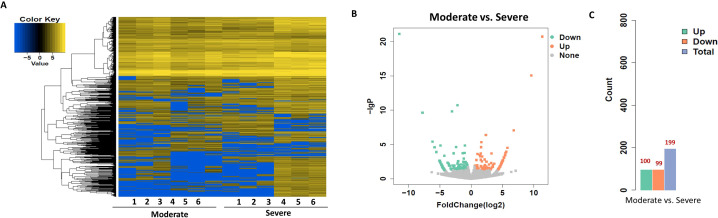
Heatmap and Volcano Plot of differentially expressed circRNAs between moderate and severe SLE groups. **(A)** The heatmap analysis revealed distinct circRNA expression profiles between the two groups. **(B)** The volcano plot shows differentially expressed circRNAs based on RNA-seq analysis. **(C)** The bar graph displays 99 downregulated circRNAs and 100 upregulated circRNAs between the two groups. N=6 each group.

To identify circRNAs associated with progression from moderate to severe SLE, differential expression and bioinformatic analyses were performed comparing six moderate and severe patients. To identify circRNAs that may serve as biomarkers distinguishing moderate from severe SLE, we performed differential expression analysis and bioinformatic analysis of circRNAs in samples from six patients with moderate and severe SLE. A heatmap was generated to illustrate the differential expression of circRNAs between the two groups, highlighting the circRNAs that were significantly upregulated or downregulated (**Figure 5**). Moreover, a volcano plot revealed a total of 199 differentially expressed circRNAs, consisting of 100 upregulated and 99 downregulated circRNAs (**Figures 5B**).

**Figure 6:**
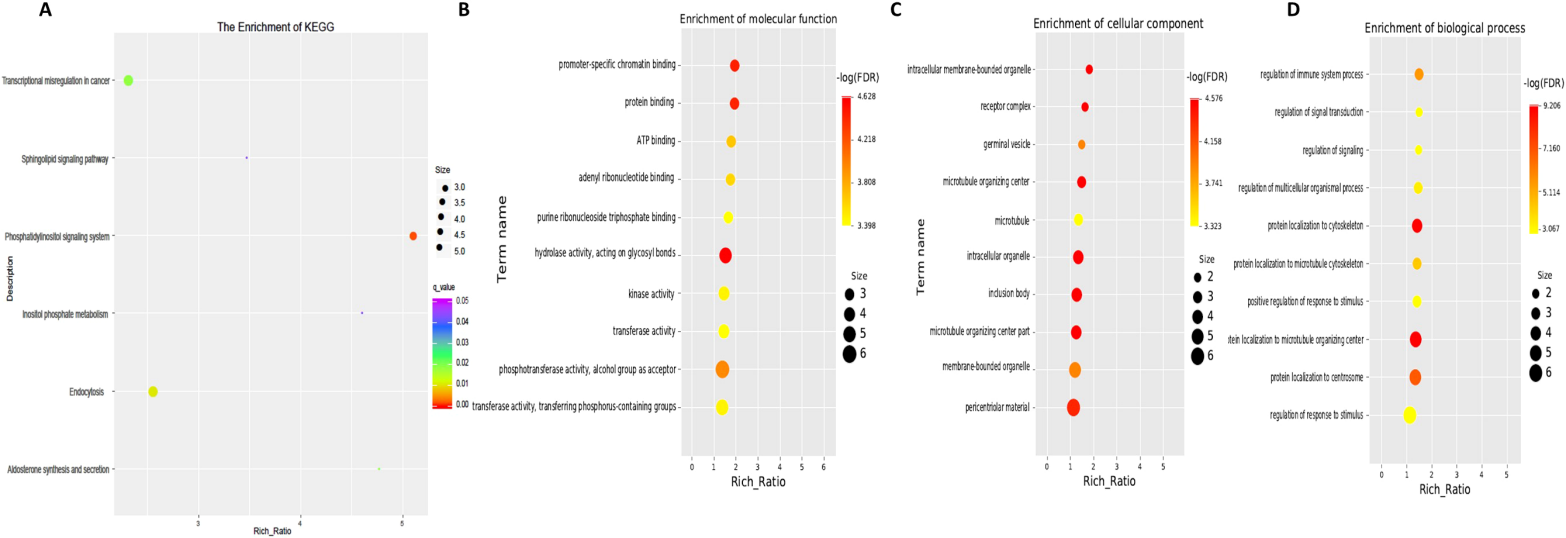
KEGG and GO Pathway enrichment analysis of differentially expressed circRNAs between moderate and severe SLE Patients. **(A)** KEGG enrichment analysis of differentially expressed circRNAs. **(B)** Molecular function GO analysis of differentially expressed circRNAs. **(C)** Cell component GO analysis. **(D)** Biological process GO analysis. N=6 each group.

Next, GO term and KEGG pathway enrichment analyses of circRNA-related genes were performed to investigate the functions associated with the dysregulated circRNAs in the moderate and severe SLE patient groups. KEGG pathway enrichment analysis of circRNA-hosting genes revealed significant differences, identifying six pathways that were enriched among the differentially expressed circRNAs. Notably, phosphatidylinositol signaling system, inositol phosphate metabolism and sphingolipid signaling pathways were among the pathways significantly enriched in these circRNAs (**Figure 6**, **Supplementary Table S5**). Gene Ontology (GO) enrichment analysis revealed 32 Molecular Function (MF) terms, 34 Cellular Component (CC) terms, and 198 Biological Process (BP) terms associated with moderate and severe SLE patients. Key enriched terms included regulation of immune system processes, ATP binding, promoter-specific chromatin binding and protein binding (**Figure 6B**, **Supplementary Tables S6**-**S8**). These findings suggest that signaling pathways, transcriptional regulation, and metabolic processes are critical to the pathogenesis of SLE, particularly when comparing moderate and severe cases.

**Figure 7:**
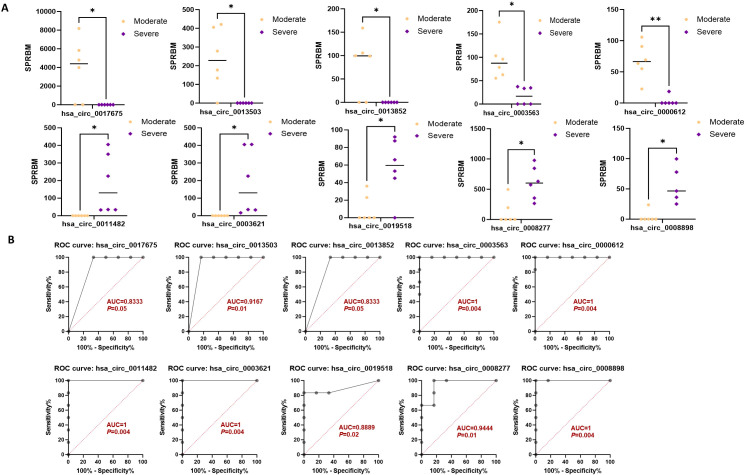
Characterization of differential expressed circRNAs in PBMCs from SLE moderate and severe patients. **(A)** Comparison of expression levels of differentially expressed circRNAs between the two groups. **(B)** ROC curve analysis of differentially expressed circRNAs in the moderate and severe groups, with AUC values displayed. N=6 each group. *P < 0.05; **P < 0.01.

Moreover, the ten circRNAs with the largest difference (5 up-regulated and 5 down-regulated) based on SPRBM are shown in **Figure 7** and summarized in **Table 3**. ROC curves were generated to assess the predictive value of the differentially expressed circRNAs we identified. Although the adjusted *P*-values for the upregulated circRNAs were not significant after correction, several showed consistent upregulation with clear fold changes and low unadjusted *P*-values. These circRNAs may still have biological relevance, especially in relation to disease severity, and deserve further validation in larger cohorts. The results demonstrated that all identified circRNAs had significant diagnostic value in distinguishing between moderate and severe SLE severity (**Figure 7**).

**Table 3:** The top ten circular RNAs (circRNAs) with the most significant expression differences (both upregulation and downregulation) between severe and moderate systemic lupus erythematosus (SLE) patients.

Gene	Up/Down	Location	Gene_name	Log2FoldChange	*P* value	*P* adjusted
hsa_circ_0017675	down	6:31271599.31356442:-	Null	-11.493	7.56E-22	2.94E-18
hsa_circ_0013503	down	2:97549419.97549614:-	ANKRD36B	-7.77129	2.34E-10	1.52E-07
hsa_circ_0013852	down	3:138570318.138572932:-	CEP70	-6.17687	3.90E-06	0.001686641
hsa_circ_0003563	down	14:101901719.101906529:+	PPP2R5C	-5.84141	2.59E-05	0.006717782
hsa_circ_0000612	down	10:45939361.45962994:-	PARGP1	-5.57524	0.0001274	0.027582013
hsa_circ_0011482	up	22:38962098.38991626:+	Null	7.113413	0.068888872	0.999873759
hsa_circ_0003621	up	14:105584133.105704086:-	Null	6.423051	0.100654936	0.999873759
hsa_circ_0019518	up	8:17268258.17286427:+	VPS37A	5.071682	0.154593886	0.999873759
hsa_circ_0008277	up	19:54633016.54697945:+	Null	5.035909	0.157100581	0.999873759
hsa_circ_0008898	up	1:161544701.161624639:-	Null	4.933635	0.208503218	0.999873759

**Figure 8:**
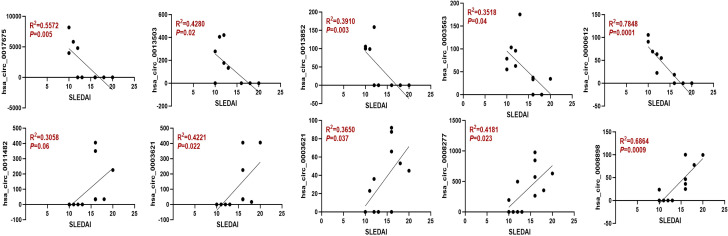
The expression of dysregulated circRNAs is closely related to the SLEDAI Score. Spearman correlation analysis was performed to show the association between differentially expressed circRNAs and SLEDAI scores in moderate and severe SLE patients. N=6 each group.

To further assess the prognostic relevance of the identified circRNAs, we analyzed the correlation between circRNA expression and the SLEDAI score. The results indicated that the expression levels of most circRNAs were significantly correlated with the SLEDAI score (*P*<0.05). However, no significant correlation was observed between the expression of hsa_circ_0011482 and the SLEDAI score (**Figure 8**).

## 4 Discussion

SLE is an autoimmune disease characterized by a complex pathogenesis and diverse clinical manifestations (17, 18). Given the complex nature of SLE pathogenesis, identifying a single, reliable biomarker for both the diagnosis and prognosis of the disease has proven to be a significant challenge, particularly for pediatric patient populations (19–21). In this study, RNA-seq was performed on peripheral blood samples from pediatric SLE patients categorized into mild, moderate, and severe groups based on SLEDAI scores, in order to identify circRNAs that could serve as prognostic markers for SLE severity. KEGG and GO analyses further identified potential pathways regulated by key circRNAs across these severity groups, suggesting possible molecular mechanisms underlying the dysregulation of these circRNAs. Additionally, through correlation analysis with the SLEDAI score and ROC curve analysis, our study identified several circRNAs as potent prognostic markers for SLE. Taken together, our data indicate that a spectrum of circRNAs could serve as valuable biomarkers for prognosis evaluation in SLE patients. Therefore, these findings offer valuable new perspectives on the diagnosis and treatment of SLE.

A wide variety of studies have demonstrated that circRNAs may represent promising biomarkers for SLE (22–24). Notably, one mechanism by which circRNAs regulate disease progression is by acting as sponges for microRNAs (miRNAs), which in turn leads to the dysregulation of gene expression levels. You et al. found that circLOC101928570 levels were significantly downregulated in SLE and showed a correlation with the systemic lupus erythematosus disease activity index. Furthermore, the downregulation of circLOC101928570 inhibited SLE progression via the miR-150-5p/c-myb/IL2RA axis. Their findings suggest that circLOC101928570 could serve as a potential biomarker for the diagnosis and treatment of SLE (22). Another study identified 18 miRNAs associated with SLE and 21 corresponding differentially expressed circRNAs by comparing T cells extracted from SLE patients and healthy controls. This led to the construction of a circRNA–miRNA–mRNA network, providing valuable insights that could contribute to the diagnosis, treatment, and prognosis of SLE patients (25). Therefore, one of our future directions would be to explore the circRNA–miRNA network across different SLE severity groups and to understand the downstream genes whose expression is influenced by these interactions.

To investigate the potential downstream activities mediated by circRNAs in SLE, we conducted KEGG pathway and GO analyses. KEGG pathway analysis revealed that the differential expression of circRNAs was associated with the ErbB signaling pathway, phosphatidylinositol signaling system, inositol phosphate metabolism, and sphingolipid signaling pathways across different SLE severity groups, suggesting that circRNAs may play a role in metabolic processes related to inositol phosphate metabolism. GO analysis indicated that differentially expressed circRNAs were involved in catalytic activity, protein binding, immune system processes, ATP binding, and promoter-specific chromatin binding. These findings suggest that circRNAs may contribute to disease pathogenesis by regulating immune responses, energy production, and transcriptional processes. Therefore, it is crucial to further explore the molecular mechanisms mediated by dysregulated circRNAs.

Our study has several limitations. First, the sample size was relatively small, with only six SLE patients per severity group providing RNA-seq samples. Given the considerable heterogeneity among patients, this limited sample size may reduce the statistical power and affect the generalizability of our findings. Future studies will aim to expand the patient cohort to validate these preliminary results and better characterize circRNA expression patterns in relation to disease severity. Second, we did not perform additional experimental validation of the differentially expressed circRNAs identified by RNA-seq. This decision was primarily due to the high cost associated with circRNA sequencing and downstream validation techniques such as qRT-PCR. Nonetheless, we acknowledge the importance of such validation and plan to confirm the expression of key candidate circRNAs in larger, independent cohorts in future research.

In conclusion, the present study identified a distinct circRNA profile in SLE across different severity groups through RNA sequencing. The results suggest that the dysregulation of circRNAs may be associated with the prognosis and pathogenesis of SLE. Thus, our work expands the understanding of the genetic landscape of SLE and provides a foundation for future research on the role of circRNAs in this disease.

## Data Availability

The data presented in this study are deposited in the NCBI Sequence Read Archive (SRA) repository under the accession number PRJNA1295701.
